# High expression of *MARVELD3* as a potential prognostic biomarker for oral squamous cell carcinoma

**DOI:** 10.3389/fgene.2022.1050402

**Published:** 2022-10-24

**Authors:** Ke Huang, Yucheng Meng, Jiyuan Lu, Lingdan Xu, Shiqi Wang, Huihui Wang, Zhaoqing Xu

**Affiliations:** ^1^ Key Laboratory of Preclinical Study for New Drugs of Gansu Province, School of Basic Medical Sciences, Lanzhou University, Gansu, China; ^2^ Key Laboratory of Dental Maxillofacial Reconstruction and Biological Intelligence Manufacturing, School of Stomatology, Lanzhou University, Gansu, China

**Keywords:** *MARVELD3*, TAMP, prognostic biomarker, oral squamous cell carcinoma, immune infiltration

## Abstract

**Objectives:** Tight junction-associated marvel proteins (TAMP) is a transmembrane protein whose members are associated with tight junctions between cells and epithelial remodeling. MARVEL domain containing 3 (*MARVELD3*) is one of the members of the TAMP. *MARVELD3*, as a novel tight junction protein involved in bicellular tight junction assembly, has attracted growing attention in the field of oncology. This study aimed to investigate the prognostic role of *MARVELD3* and to determine how it functions in tumorigenesis in oral squamous cell carcinoma (OSCC), thus providing additional data to help the guidance of clinical practice.

**Materials and Methods:** RNA-seq data and relevant clinical information were obtained from TCGA. Bioinformatics means used in this study included differential gene expression analysis, KM survival curve analysis, univariate and multivariate Cox regression analyses, nomogram analysis, ROC curve analysis, methylation level analysis, gene function enrichment analysis, and immune cell infiltration analysis.

**Results:** *MARVELD3* was significantly higher expressed in OSCC tissue than in normal tissue, and the overall survival of the high expression group was significantly lower than that of the normal group. Univariate and multivariate Cox regression analyses showed that *MARVELD3* could serve as an independent contributing factor to poor OSCC prognosis. The nomograms and ROC curves supported the results above. Its expression was negatively correlated with DNA methylation sites. Analysis of PPI networking and gene functional enrichment showed that *MARVELD3* was involved in the functional activities of DNA and RNA and was associated with immune cell infiltration.

**Conclusion:** The high expression of *MARVELD3* is associated with poor prognosis in OSCC, and *MARVELD3* could be recognized as a novel independent prognostic factor for OSCC.

## Introduction

Oral squamous cell carcinoma (OSCC) is the most prevalent oral malignant tumor worldwide, with a five-year survival rate of only 50% (Manzano-Moreno et al., 2021). At present, the main treatment methods for OSCC are mainly surgery, chemotherapy, and radiotherapy. The major causes of high mortality are high tumor invasion, lymph node involvement, poor response to therapy, and early local recurrence (Cierpikowski et al., 2021). To be able to effectively improve the survival rate and improve prognosis in patients with OSCC, there is an urgent need to look for a potential biomarker. This marker can be used as a diagnostic indicator as well as a prognostic indicator. The Tight junction Associated Marvel Proteins (TAMP) are transmembrane proteins. The TAMP family consists of occludin, tricellulin (also called *MARVELD2*), and MARVEL domain containing 3 (*MARVELD3*) (Heymans et al., 2021). Its members are associated with tight junctions between cells and epithelial remodeling. Most studies focus on the connection function between epithelial cells and the role of epithelial mesenchymal cells in transformation (Kojima et al., 2011). Studies have confirmed that tricellulin is identified as the first marker of tricellular tight junction in epithelial cells. Its loss affects the tight binding of the tricellular tight junction as well as the barrier function of the epithelial cells (Ikenouchi et al., 2005; Ikenouchi et al., 2008). At the same time, Steed et al. found that normal expression of *MARVELD3* is not necessary for the formation of functionally tight junctions, but it is a decisive factor in epithelial paracellular permeability properties (Steed et al., 2009). Some literature has also found that *MARVELD3* is involved in the process of promoting cell migration by EMT in hepatocellular carcinoma cells, and it inhibits the occurrence and progression of this process through the NF-κB signaling pathway (Li et al., 2021). Some scholars have shown that *MARVELD2* and *MARVELD3* are included in the genes that are highly correlated with the close connection of epithelial tumor cells (Kohn et al., 2014). In addition to this, studies have shown that *MARVELD3* has two isoforms and has a broad tissue distribution. *MARVELD3* functions as a regulator of epithelial cell proliferation, migration, and survival in human colon and pancreatic cancer cells, but the role it plays in OSCC is unclear (Li et al., 2021). In addition to that, previous studies have found that MARVEL domain-containing 1 (*MARVELD1*) could inhibit tumor cell proliferation and enhance the sensitivity to chemotherapeutic drugs in hepatocellular carcinoma (Zhang et al., 2019). So, we suspect that *MARVELDs* may have a different expression in OSCC than in normal tissue. With the development of bioinformatics analysis technology and the establishment and improvement of various databases, we have studied this problem. This study conducted in-depth analysis of *MARVELDs* expression in OSCC and assessed their potential value as prognostic biomarkers, providing a new method for guiding clinical work and effectively and accurately assessing the long-term prognosis of OSCC patients.

## Materials and methods

### Data collection and processing

The Cancer Genome Atlas (TCGA) database was utilized to collect the data on gene expression profiles of 329 samples with OSCC and 32 non-OSCC normal tissue samples. The RNA-seq data and the corresponding clinical information were downloaded from TCGA. Transcripts per million reads (TPM) format of RNA-seq data (from TCGA), through Toil process standardization, were downloaded from UCSC XENA (https://xenabrowser.net/datapages/). Log2 fold change (log2FC) was calculated to further compare mRNA expression levels between tumor and normal samples.

### Survival and statistical analyses

Through data analyses, the samples were divided into high-expression and low-expression groups according to the median expression level of *MARVELDs*. At the same time, Kaplan-Meier survival analysis was performed using the R package (survive, version 0.4.9 and survival, version 3.2.10). The KM curve was plotted to effectively assess the relationship between the expression level of *MARVELDs* and the overall survival of patients.

### Univariate and multivariate Cox regression analyses

To investigate whether the expression level of *MARVELD3*, gender, age, tumor stage (T stage), lymphatic involvement grade (N stage), lymph vascular invasion, and histologic grade were independent risk factors for oral squamous cell carcinoma, univariate and multivariate Cox regression analyses were utilized. We used the R package (survival, version 3.2.10) for data processing and set Hazard ratios (HR) and 95% confidence intervals. The significance threshold was set as *p* < 0.05.

### Construction of nomograms and ROC curves

Constructing nomograms were allowed to predict the prognosis of patients with oral squamous cell carcinoma based on age, gender, TNM, clinical grade, and *MARVELD3* expression levels. The ROC curves were plotted using the R package (pROC, version 1.17.0.1 and ggplot2, version 3.3.3) to assess the diagnostic value of *MARVELD3*.

### *MARVELD3* methylation level analysis

For the gene methylation data, we used Illumina Human Methylation 450 array methylation chip data and the RNA-Seq data of the level 3 HTSeq-FPKM format downloaded from the Head and Neck Squamous Cell Carcinoma (HNSC) project in TCGA. Data with no clinical information were discarded. Samples that belonged to oral cancer sites (such as alveolar ridge, base of tongue, buccal mucosa, floor of mouth, hard palate, oral cavity, and oral tongue) were retained, while others (such as hypopharynx, larynx, lip, oropharynx, and tonsil) were discarded. Methylation results were visualized using the R package (ggplot2, version 3.3.3).

### *MARVELD3*-related gene function enrichment analysis

Statistical analysis of gene-gene correlations was performed using spearman correlation by the R package (stat, version 3.6.3). |Cor spearman|≥0.4 and p-spearman<0.05 were set as the filter criteria. Besides, the GeneMANIA (http://genemania.org) and the STRING database (https://cn.string-db.org/) (Szklarczyk et al., 2019) were used to analyze the protein-protein interaction (PPI) networks and to look for relevant signaling pathways and functional similarities. Based on the top 50 relevant genes found, the R package (org.Hs.eg.db, version 3.10.0 and clusterProfiler, version 3.14.3) (Yu et al., 2012) were used for Gene Ontology (GO) and Kyoto Encyclopedia of Genes and Genomes (KEGG) analysis to screen and evaluate potential gene functions correlated with *MARVELD3*.

### Immune cell infiltration analysis

In this study, the R package (GSVA package, version 1.34.0) (Hänzelmann et al., 2013) was used to analyze the 24 types of immune cells in OSCC.

## Results

### Clinical characteristics of oral squamous cell carcinoma

There were 329 primary OSCC samples and 32 normal samples downloaded from TCGA. Clinical information includes tumor stage (T stage), lymphatic involvement grade (N stage), distant metastases grade (M stage), clinical stage, radiation therapy, primary therapy outcome, gender, race, age, histologic grade, anatomic tumor subdivision, smoker, alcohol history, lymphovascular invasion, lymphnode neck dissection, overall survival (OS) event, disease special survival (DSS) event, and progress free interval (PFI) event (Table 1).

**TABLE 1:** Clinical characteristics of OSCC patients.

Characteristic	Levels	Overall	Characteristic	Levels	Overall
n		329	Histologic grade, n (%)	G1	52 (16.2%)
T stage, n (%)	T1	18 (5.6%)		G2	200 (62.3%)
	T2	105 (32.9%)		G3	67 (20.9%)
	T3	82 (25.7%)		G4	2 (0.6%)
	T4	114 (35.7%)	Anatomic neoplasm subdivision, n (%)	Alveolar Ridge	18 (5.5%)
N stage, n (%)	N0	168 (53.3%)		Base of tongue	23 (7%)
	N1	56 (17.8%)		Buccal Mucosa	22 (6.7%)
	N2	88 (27.9%)		Floor of mouth	61 (18.5%)
	N3	3 (1%)		Hard Palate	7 (2.1%)
M stage, n (%)	M0	310 (99.4%)		Oral Cavity	72 (21.9%)
	M1	2 (0.6%)		Oral Tongue	126 (38.3%)
Clinical stage, n (%)	Stage I	11 (3.4%)	Smoker, n (%)	No	87 (26.9%)
	Stage II	79 (24.8%)		Yes	236 (73.1%)
	Stage III	65 (20.4%)	Alcohol history, n (%)	No	105 (32.7%)
	Stage IV	164 (51.4%)		Yes	216 (67.3%)
Radiation therapy, n (%)	No	116 (39.3%)	Lymphovascular invasion, n (%)	No	164 (68.6%)
	Yes	179 (60.7%)		Yes	75 (31.4%)
Primary therapy outcome, n (%)	PD	35 (12.6%)	Lymphnode neck dissection, n (%)	No	45 (13.8%)
	SD	4 (1.4%)		Yes	282 (86.2%)
	PR	3 (1.1%)	OS event, n (%)	Alive	179 (54.4%)
	CR	236 (84.9%)		Dead	150 (45.6%)
Gender, n (%)	Female	102 (31%)	DSS event, n (%)	Alive	219 (70.2%)
	Male	227 (69%)		Dead	93 (29.8%)
Race, n (%)	Asian	9 (2.8%)	PFI event, n (%)	Alive	194 (59%)
	Black or African American	21 (6.6%)		Dead	135 (41%)
	White	288 (90.6%)	Age, median (IQR)		61 (54, 70.25)
Age, n (%)	≤60	155 (47.3%)			
	>60	173 (52.7%)			

### *MARVELD* expression in oral squamous cell carcinoma patients

**FIGURE 1:**
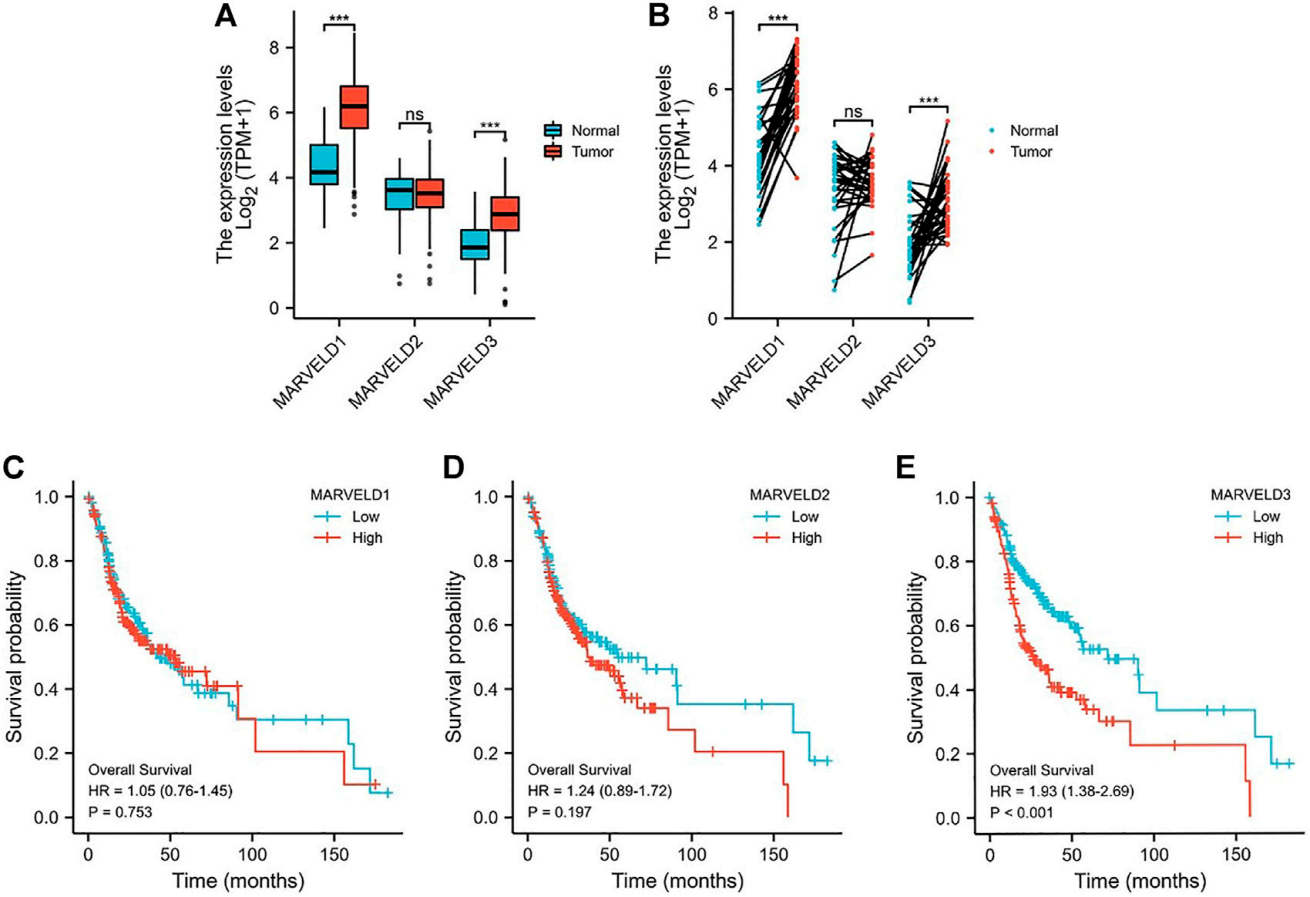
The MARVELDs expressions and survival analysis in OSCC. **(A)** Differential expression analysis of MARVELDs in non-paired samples of OSCC patients. **(B)** Differential expression analysis of MARVELDs in paired samples of OSCC patients. **(C–E)** K-M survival curves of the associations between MARVELDs expressions and overall survival.

Through the analyses of the data from the TCGA database, the results showed that the expression levels of *MARVELD1* and *MARVELD3* were significantly higher than those of normal tissues (Figures 1A,B). There was no statistical difference in the expression level of *MARVELD2* compared to normal tissue. Then, through KM survival analysis, the relationships between the expression levels of *MARVELDs* and the overall survival of OSCC patients were analyzed. According to the KM survival curves, the high expression of *MARVELD3* was significantly associated with the worse overall survival in OSCC patients (Figure 1E). There were no significant correlations between the expression levels of *MARVELD1* and *MARVELD2* with worse overall survival (Figures 1C,D). The results suggested that the high expression of *MARVELD3* was associated with the poor prognosis of OSCC and that *MARVELD3* may be considered an oncogene for OSCC.

### Diagnostic value of *MARVELD3* in oral squamous cell carcinoma

**FIGURE 2:**
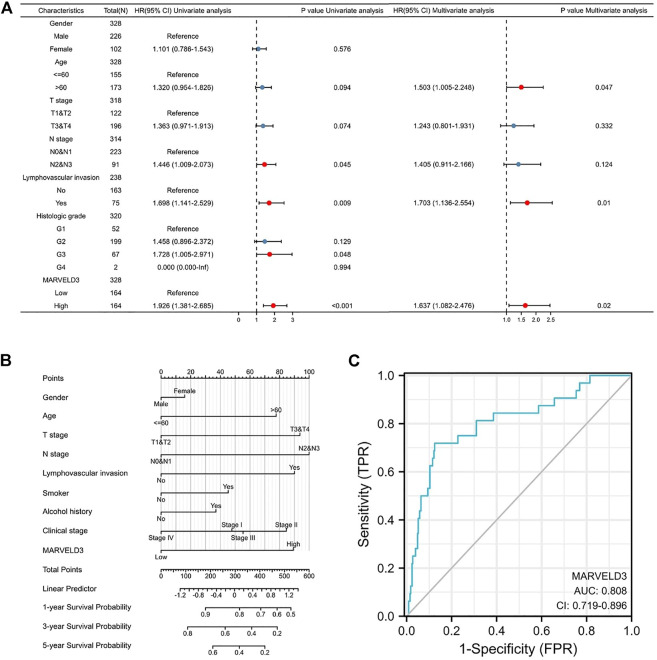
Diagnostic value of MARVELD3 in OSCC. **(A)** Univariate and multivariate Cox regression analyses. HR > 1 indicates disadvantageous factors, and HR < 1 indicates protective factors. Red dots are risk factors. **(B)** The nomograms were developed by integrating the MARVELD3 expression with key clinical characteristics. **(C)** The diagnostic value of MARVELD3 expression was evaluated using the ROC curve.

Based on the results above, we selected *MARVELD3* for further in-depth analysis. The results of the univariate Cox regression analysis showed that the high expression of *MARVELD3* was a contributing factor to the poor prognosis of OSCC. The results of multivariate Cox regression analysis showed that the high expression of *MARVELD3* was an independent prognostic factor for poor OSCC prognosis (Figure 2A). In addition, to be able to predict the survival probability of patients at 1, 3, and 5 years, we constructed nomogram analysis including factors such as age, gender, TNM stage, clinical stage, etc. (Figure 2B). At the same time, we used the ROC curves to evaluate the diagnostic value of *MARVELD3* (Figure 2C). The result of the ROC curves showed that the area under the curve (AUC) of *MARVELD3* was 0.808, which indicated a considerable diagnostic value. Combined with the above analyses, *MARVELD3* may be an unfavorable factor for survival in OSCC patients and an independent predictor of poor prognosis for OSCC.

### *MARVELD3* methylation level analysis

**FIGURE 3:**
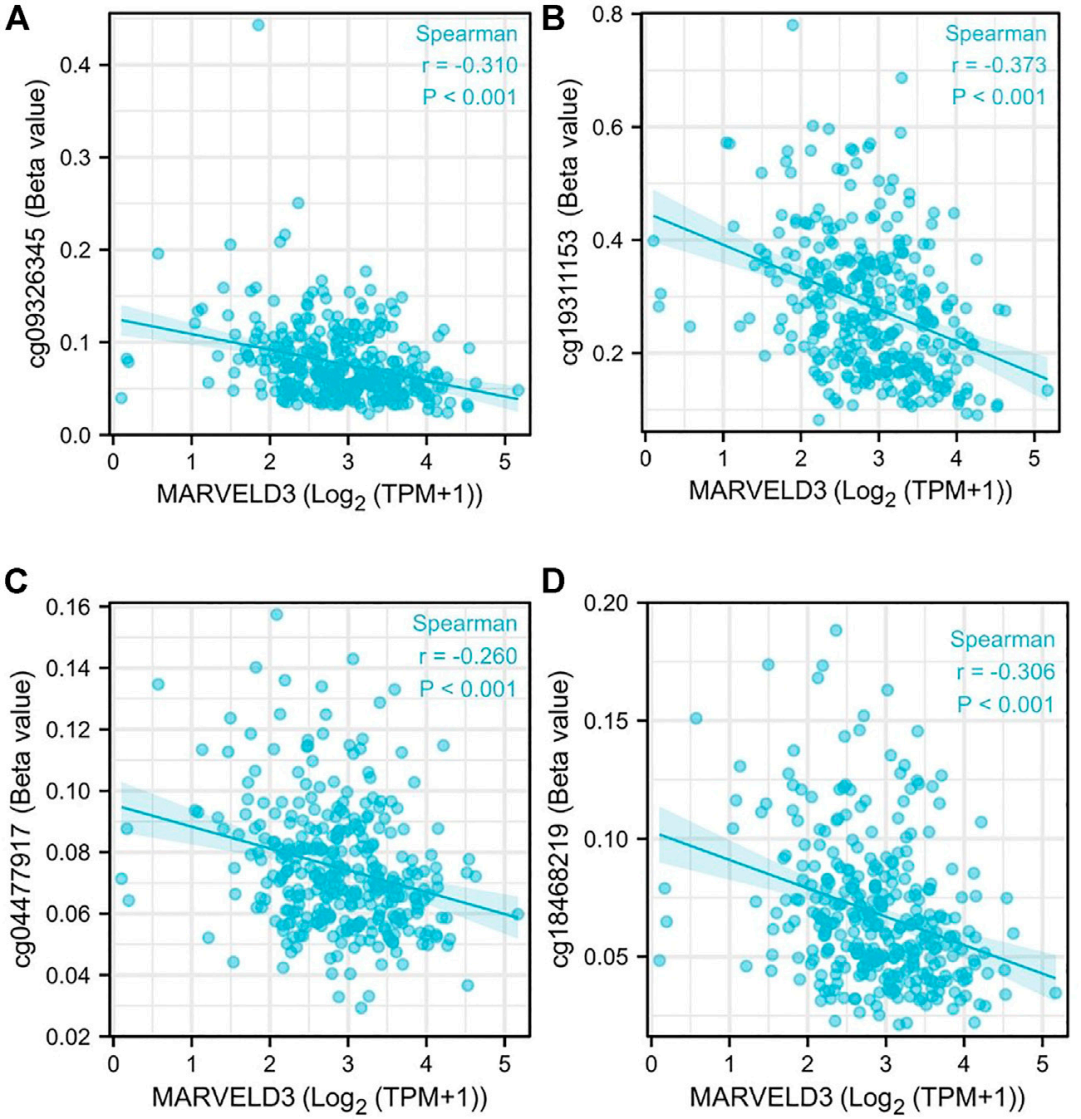
*MARVELD3* methylation level analysis. **(A–D)** Correlation of *MARVELD3* expression with methylation sites of cg09326345, cg19311153, cg004477917, and cg18468219.

Our analysis showed that the DNA methylation levels were inversely correlated with the expression of *MARVELD3* in four methylation sites (cg09326345, cg19311153, cg004477917, and cg18468219) (Figure 3).

### *MARVELD3* correlation interactive networks analysis

**FIGURE 4:**
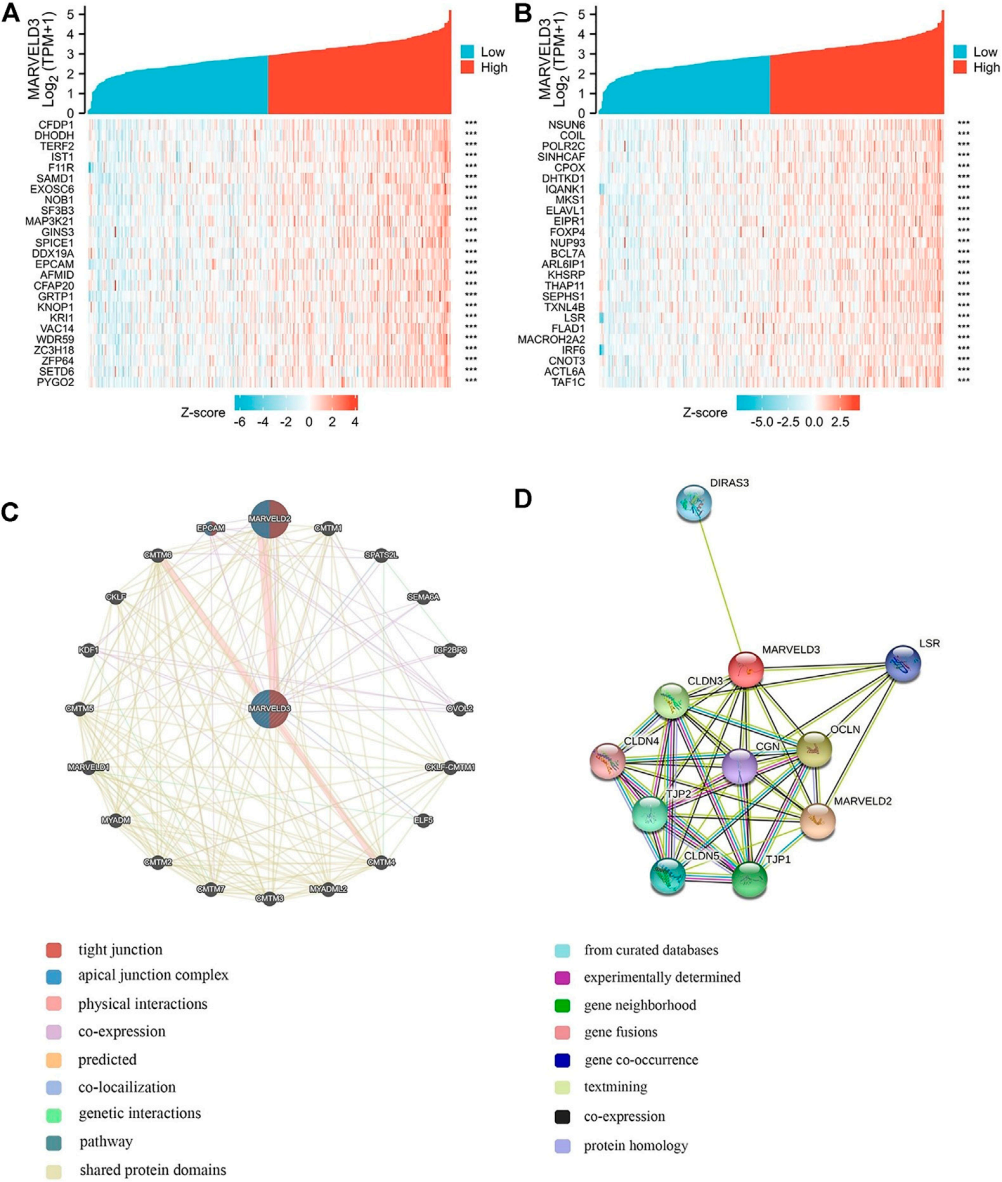
MARVELD3 correlation interactive networks. **(A–B)** Top 50 genes related to MARVELD3 expression. **(C)** Network diagram of 20 genes associated with MARVELD3. **(D)** Protein-protein interaction (PPI) network analysis of 11 interacting proteins correlated with MARVELD3.

Gene correlation analyses were performed and the top 50 relevant genes were presented (Figures 4A,B). In addition, PPI network analysis was plotted using the GeneMANIA database and the STRING database (Figures 4C,D). Among them, the related proteins are mainly related to the tight junction, leukocyte transendothelial migration, and cell adhesion molecules.

### Functional enrichment analysis of *MARVELD3*

**FIGURE 5:**
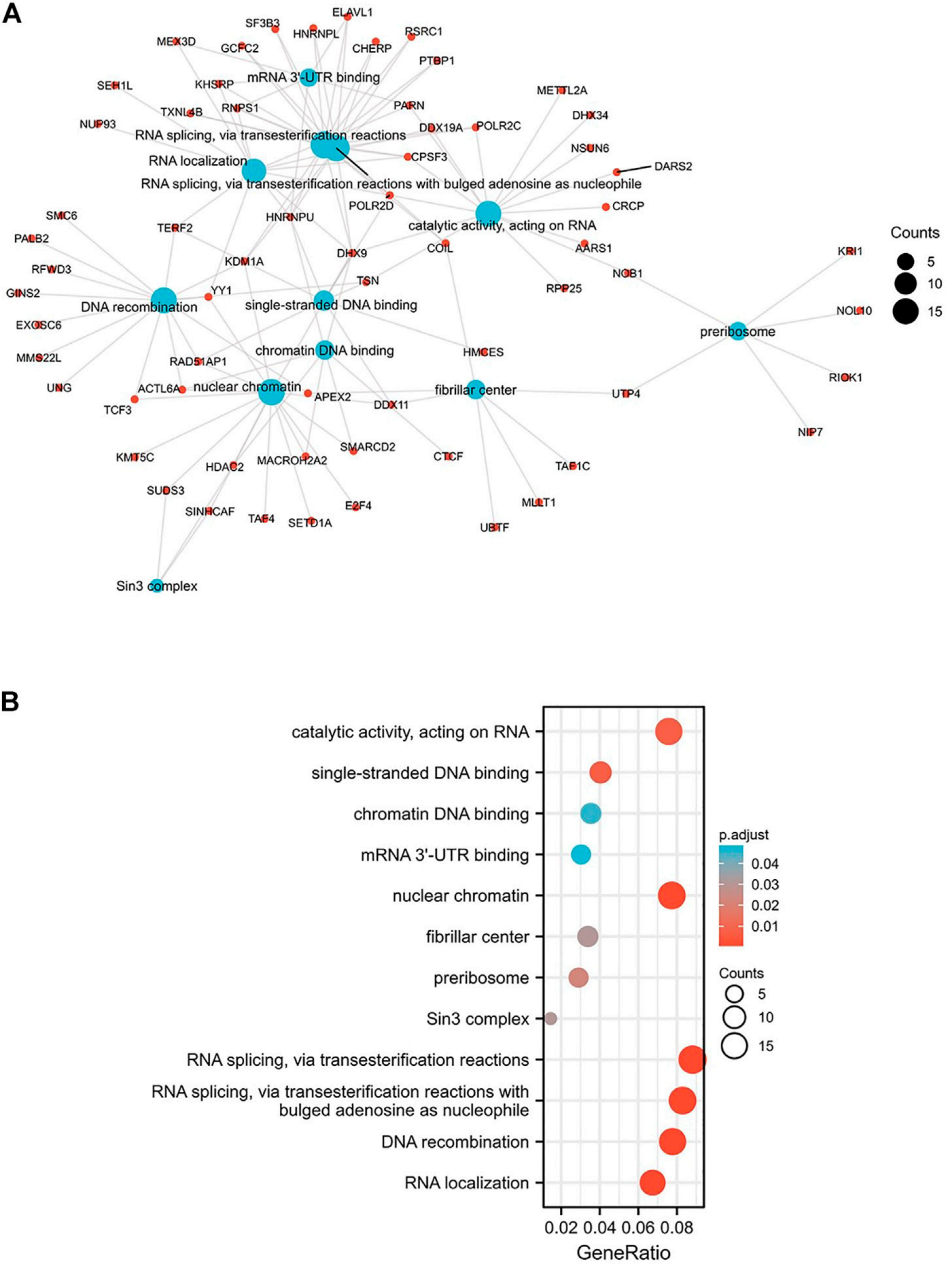
GO/KEGG enrichment analysis. **(A)** Network visualization of GO/KEGG enrichment analysis. **(B)** Bubble plot of GO/KEGG enrichment analysis.

The biological functions of the genes associated with the expression of *MARVELD3* were analyzed by GO and KEGG. The results showed that there was a range of functions related to the expression of *MARVELD3*, such as RNA splicing *via* transesterification reactions, RNA splicing *via* transesterification reactions with bulged adenosine as nucleophile, DNA recombination, RNA localization, nuclear chromatin, and catalytic activity acting on RNA, etc. (Figures 5A,B).

### The correlation between *MARVELD3* expression and immune cell infiltrations in oral squamous cell carcinoma

**FIGURE 6:**
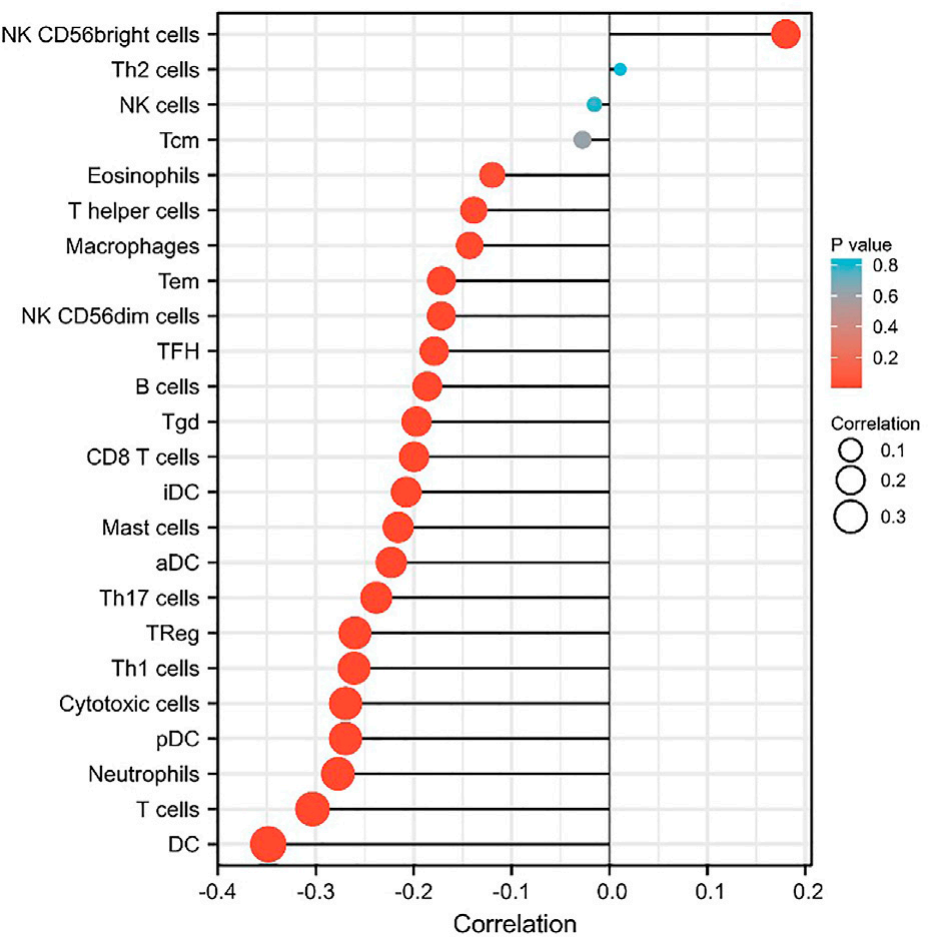
The correlation of *MARVELD3* with 24 types of immune cells in OSCC.

Based on the above results, we further explored the relationship between the *MARVELD3* expression and immune cell infiltration. Data downloaded from TCGAs related to 24 types of immune cells. The results showed that the expression level of *MARVELD3* was inversely correlated with the infiltration level of various immune cells such as B cells, T cells, and DC cells (Figure 6).

## Discussion

OSCC is a common malignancy in the human oral cavity, and its prognosis is mostly poor, which significantly affects the overall survival rate of patients. The incidence tends to increase year by year. The clinical outcome and prognosis of OSCC remains dismal; more than 50% of patients die of this disease or complications within 5 years (Lo et al., 2003). Lip, oral cavity, and oropharynx combined were responsible for about 447,751 new cancer cases with an estimated 228,389 deaths in 2018, which accounts for 2.4% of all cancer deaths. In addition to this, head and neck cancer is the fourteenth in terms of incidence but the thirteenth in terms of mortality (WHO, 2020). It is important to look for a potential biomarker that can diagnose, predict the prognosis of OSCC, and serve as a therapeutic target.

In this study, we found that the expressions of *MARVELD1* and *MARVELD3* were significantly higher in OSCC than in normal tissues. Further studies found that high expression of *MARVELD3* correlated significantly with poor overall survival in patients. In addition, univariate and multivariate Cox regression analyses showed that *MARVELD3* could be served as an independent predictor of poor prognosis for OSCC. The clinical predictive and diagnostic value of *MARVELD3* were further evaluated by the nomograms and the ROC curve, and the results also confirmed the potential value of *MARVELD3*. Moreover, *MARVELD3* was inversely correlated with methylation sites, which was consistent with the characteristics of the oncogene.

Members of the TAMP family are predominantly transmembrane proteins. The functions of transmembrane proteins are ensuring the interaction of tight junctions (TJs) strands between adjacent cells (Heymans et al., 2021). The major TJ proteins are classified according to their physiological role in enabling or preventing paracellular transport. *MARVELD3* is linked to a multitude of TJ-associated regulatory and scaffolding proteins (Günzel and Fromm, 2012). By exploring the associated gene functions of *MARVELD3* in OSCC, the results suggested that its underlying biological function may be related to RNA splicing *via* transesterification reactions, RNA splicing *via* transesterification reactions with bulged adenosine as nucleophile, DNA recombination, RNA localization, nuclear chromatin, and catalytic activity acting on RNA. Through PPI network analysis, we found that *MARVELD3* and the claudins (CLDNs) family were closely related. CLDNs are a family of at least 27 transmembrane proteins (Krause et al., 2008; Bhat et al., 2020). Structurally and functionally, CLDNs are commonly used for intercellular adhesion, maintaining cell polarity, and playing a role in barrier function (Hashimoto and Oshima, 2022). Alternatively, overexpression of CLDNs has also been reported to increase aberrant localization and function in gastric, lung, prostate, ovarian, colorectal, and breast cancers, promoting metastasis and progression (Tabariès and Siegel, 2017). These evidences further validated the research value of *MARVELD3* high expression in tumor tissues. Coincidentally, in the squamous epithelium of the oral cavity, cancer may occur when the dynamic structure of TJs localized in its tissues changes. At the same time, scholars have studied that the loss of claudin-7 (CLDN7) expression is associated closely with invasion and lymph metastasis. It is an unfavorable prognostic factor in patients with OSCC (Yoshizawa et al., 2013). This finding further illustrates the abnormality in the expression of genes associated with cell tight junctions in OSCC, thus providing evidence for our research.

The results of immune cell infiltration analysis showed that *MARVELD3* was inversely correlated with a variety of immune cells including DCs, T cells, neutrophils, and B cells. Studies have reported similar situations (Li et al., 2016). We speculate that this phenomenon may be explained by the inability of the immune system in OSCC patients to recognize *MARVELD3* enough. It cannot be recognized as a reliable antigen and aggregated to it. At the same time, this also meant that with the upregulation of *MARVELD3* expression, OSCC would not have a strong host immune response. Interestingly, according to our findings, the expression of *MARVELD3* was positively correlated with NK CD56^bright^ cells. Natural cytotoxicity, mediated by natural killer (NK) cells plays an important role in the inhibition and elimination of malignant tumor cells (Koo et al., 2013). The phenotype of NK cells is defined by their CD56 expression and lack of CD3 expression, of which CD56^bright^ and CD56^dim^ subpopulations can be divided according to the membrane density of CD56. CD56^bright^ cells mediate low cytotoxicity, CD56^dim^ mainly exerts strong cytotoxicity, and CD56^bright^ may be a precursor to CD56^dim^ (Cooper et al., 2001; Bauernhofer et al., 2003; Watanabe et al., 2010; Dowell et al., 2012). We speculated that this may be that the high expression of *MARVELD3* stimulates the conversion of CD56^bright^ to CD56^dim^, resulting in the phenomenon that *MARVELD3* is positively correlated with it. But still, the mechanism by which *MARVELD3* is negatively correlated with immune cell infiltration needs to be further studied. Anyway, the relationship between the high expression of *MARVELD3* and the clinical characteristics of OSCC obtained in this study may provide evidence for such studies. For OSCC patients in the process of postoperative recovery, doctors can detect the expression of prognostic markers such as *MARVELD3* to timely grasp their situation and make correct and reasonable treatment measures, so as to effectively improve the survival status of OSCC patients. However, there are limitations to our study. The underlying molecular mechanism of MARVELD3 in OSCC immune infiltration has not been thoroughly studied. Meanwhile, the reason and mechanism of MARVELD3 overexpression in OSCC have not been thoroughly studied, and the reason for the differential expression between MARVELD1,2,3 is not clear. Therefore, more research needs to be included to explore the underlying molecular mechanism of MARVELD3 in OSCC. Therefore, more researches are needed in the future and our research group will continue to focus on this topic.

## Conclusion

In conclusion, our preliminary findings revealed that the high expression of *MARVELD3* was strongly and positively correlated with the poorer prognosis in OSCC, and *MARVELD3* could serve as a novel independent prognostic factor in OSCC.

## Data Availability

The raw data supporting the conclusions of this article will be made available by the authors, without undue reservation.
